# Community Health Workers Equipped with an mHealth Application Can Accurately Diagnose Hypertension in Rural Guatemala

**DOI:** 10.5334/gh.1423

**Published:** 2025-04-17

**Authors:** Sean Duffy, Taryn McGinn Valley, Alejandro Chavez, Valerie Aguilar, Juan Aguirre Villalobos, Kaitlin Tetreault, Guanhua Chen, Elizabeth White, Alvaro Bermudez-Cañete, Do Dang, Julie Cornfield, Yoselin Letona, Rafael Tun

**Affiliations:** 1 University of Wisconsin-Madison School of Medicine and Public Health Madison, Wisconsin, US; 2 Department of Anthropology, University of Wisconsin-Madison, Madison, WI, US; 3 Department of Family Medicine and Community Health, University of Wisconsin School of Medicine and Public Health, Madison, WI, US; 4 Department of Family & Community Medicine, Northwestern University Feinberg School of Medicine, Chicago, IL, US; 5 Department and Medical Informatics, University of Wisconsin School of Medicine and Public Health, Madison, WI, US; 6 Department of Biostatistics and Medical Informatics, University of Wisconsin School of Medicine and Public Health, Madison, WI, US; 7 Operations Research Group, University of Toronto, Toronto, ON, CA; 8 Stanford University, Stanford, US; 9 Department of Family Medicine, University of Maryland Medical Center, Baltimore, MD, US; 10 Department of Foreign Languages, Federal University of Sergipe, Sergipe, BR; 11 Hospital Obras Sociales Monseñor Gregorio Schaffer, San Lucas Tolimán, GT

**Keywords:** Hypertension, Guatemala, community health workers, mHealth, task sharing

## Abstract

**Background:**

Hypertension is a leading global cause of morbidity and mortality and is increasing in low- and middle-income countries, where unawareness of hypertension is a primary obstacle to management. Community health workers (CHWs) in combination with mobile health (mHealth) tools are increasingly used in LMIC health systems to strengthen primary care infrastructure. In this study, we applied this care model to hypertension in rural Guatemala by comparing the accuracy of CHWs equipped with an mHealth clinical decision support application in diagnosing hypertension to concurrent physician evaluation.

**Methods:**

We performed a prospective diagnostic accuracy study in which adults from rural Guatemalan communities were assessed independently by a CHW aided by a mHealth application and a physician. Assessment included medical history; measurement of blood pressure, height and weight; and determination of hypertension status. CHW-physician agreement on hypertension status and past medical history elements was assessed by Kappa analysis and proportional agreement, with *a priori* thresholds of Kappa = 0.61 and agreement of 90%. Agreement on patient measurements was evaluated using Bland-Altman and regression analyses.

**Results:**

Of 359 participants enrolled, 47 (13%) were confirmed to have hypertension and another 11 (3%) had possible hypertension. CHW-physician agreement was high for hypertension diagnosis, with Kappa = 0.8 (95% CI = 0.72, 0.88) and overall agreement 92.8% (95% CI = 90.1%, 95.4%). Bland-Altman analysis showed small biases toward lower systolic blood pressure, higher height, and lower BMI measurements by CHWs. Most patient history characteristics showed moderate to almost perfect (Kappa: 0.41–1) agreement between physicians and CHWs.

**Conclusions:**

In this study based in rural Guatemala, CHWs using a mHealth clinical decision support application were found to screen adult patients for hypertension with similar accuracy to a physician. This approach could be adapted to other low-resource settings to reduce the burden of undiagnosed and untreated hypertension.

## Introduction

Hypertension is the most common chronic disease in adults, and a leading global cause of morbidity and mortality. Over the past 30 years, the number of people living with hypertension has doubled, from 650 million to 1.3 billion (1). Most of this increase has occurred in low- and middle-income countries (LMIC), where more than three quarters of patients with hypertension now live (1). While most patients can effectively manage their hypertension with lifestyle changes and inexpensive medications, a primary obstacle to hypertension control in LMIC is unawareness of the disease: less than 40% of patients in LMIC have been diagnosed (2). This problem is particularly acute in rural areas, where fewer than one in three patients with hypertension have been diagnosed (3).

Inadequate primary care infrastructure and healthcare workforce constraints impede the identification and management of chronic, non-communicable diseases like hypertension in LMIC (4). To build LMIC health systems’ capacity to address both acute and chronic health concerns, task-sharing with a variety of lay health workers (LHWs), such as community health workers (CHWs), has emerged as a viable strategy (5,6,7,8). In addition, mobile health (mHealth) tools—applications designed for smartphones and tablets and used at the point-of-care—have shown promise for enhancing health worker diagnostic accuracy, increasing adherence to treatment protocols and reducing required training times (9,10,11).

Previous studies suggest that LHWs can accurately screen for hypertension and other cardiovascular risk factors in LMIC (12,13,14). However, these prior studies had methodological limitations, including lack of concurrent diagnostic assessment by a “gold-standard” evaluator (such as a physician), no *a priori* definition of a goal diagnostic agreement metric to allow for appropriate hypothesis testing, and use of simple correlation to compare blood pressure (BP) measurements between health workers rather than more robust analyses of measurement agreement. In addition, while prior work has shown that LHWs using automatic BP monitors have improved accuracy compared to physicians using manual cuffs (15), there have been no comparisons of BP measured concurrently by LHWs and physicians with both using automated monitors, the current standard of care (16). Finally, previous studies have not evaluated the impact of mHealth tools on LHW accuracy for hypertension diagnosis compared to physician diagnosis. With this study in rural Guatemala, we aimed to fill gaps in the existing literature and further the evidence for task-sharing for hypertension care in LMIC by rigorously assessing the accuracy of CHWs equipped with a novel mHealth clinical decision support application in diagnosing hypertension compared to concurrent physician evaluation.

## Methods

### Study design, setting, and participants

We conducted a prospective diagnostic accuracy study comparing hypertension diagnosis performed by CHWs aided by a clinical decision support application and diagnosis by a physician. The study was carried out in the rural communities of San Lucas Tolimán (SLT), a municipality in the Western Highlands region of Guatemala. San Lucas Tolimán was chosen as the site for this study because the Guatemalan institutional member of our collaboration, the San Lucas Mission, serves these communities. The members of these communities predominantly belong to the Indigenous Kaqchikel Maya ethnic group. This study was reviewed and approved by the University of Wisconsin IRB (#2021-1462, initial approval 11/30/2021), as well as by the San Lucas Mission Healthcare Committee (initial approval 09/19/2020).

Eligibility criteria included 18 years or older, not currently pregnant, and able to tolerate an upper arm BP measurement. CHWs recruited participants via word of mouth in their respective communities (convenience sampling). Study activities were carried out in a central location in each community, generally a communal building used for mobile clinics and other public events. Study activities occurred between June 2 and December 8, 2022.

We used the STARD guidelines (17) for the conduct and reporting of diagnostic accuracy studies (see Appendix 1 for STARD checklist).

### Test methods

#### Description of index and reference tests

The index (experimental) test for this study was CHW diagnosis of hypertension. The CHWs who carried out this study are affiliated with a local non-profit organization that provides health care and other social services. Individuals are recruited for this CHW program from the rural communities of San Lucas Tolimán. Basic requirements for participation in the CHW program include fluency in Spanish and Kaqchikel (the predominant local Mayan language), as well as the ability to read and write. Initial training for CHWs consists of one weekend per month for three years, focusing on health education and promotion.

We developed an mHealth application on the CommCare platform (Dimagi, Cambridge, MA) to assist CHWs in the diagnosis of hypertension. CommCare runs on mobile devices using the Android operating system (this study utilized Android tablets). The application functions offline, with data uploaded to a secure centralized server when internet is available. Similar to an application designed by our group to provide clinical decision support to CHWs for diabetes care (18), this tool uses branching logic to guide users through patient evaluation—including collecting basic demographic information, recording past medical history and current medications, recording social history, measuring blood pressure and other vital signs—and to provide guidance regarding hypertension status. The social history focuses on factors known to influence blood pressure, including alcohol use, tobacco use, physical activity, and exposure to cookstove air pollution (19,20).

The CHWs who participated in this study were experienced in using CommCare applications for other clinical initiatives administered by the CHW program, including childhood nutrition monitoring and diabetes care. These CHWs received 12 hours of additional training prior to this study focused on hypertension basic pathophysiology and diagnosis; measurement of BP, height, and weight; and use of the mHealth application. Each CHW also successfully completed a skills evaluation involving a simulated study encounter, including use of the application, prior to participating in the study.

Physician diagnosis of hypertension served as the reference standard. We chose this reference standard because medical diagnosis is still most commonly the domain of physicians and other advanced clinicians (21). Two Guatemalan physicians and one American physician participated in the study. All physicians had extensive prior experience working with this population. One of the Guatemalan physicians was fluent in Spanish and Kaqchikel, and one Guatemalan physician and the American physician were fluent in Spanish (assisted by a Spanish–Kaqchikel interpreter when necessary). All physicians received training in diagnostic definitions of hypertension, BP measurement, and a physician-specific version of the mHealth application described above, which they used for study data collection. The physician-specific version of the application differed primarily from the CHW version in that it provided less specific guidance on patient history taking.

#### Hypertension diagnostic algorithm

**Figure 1:**
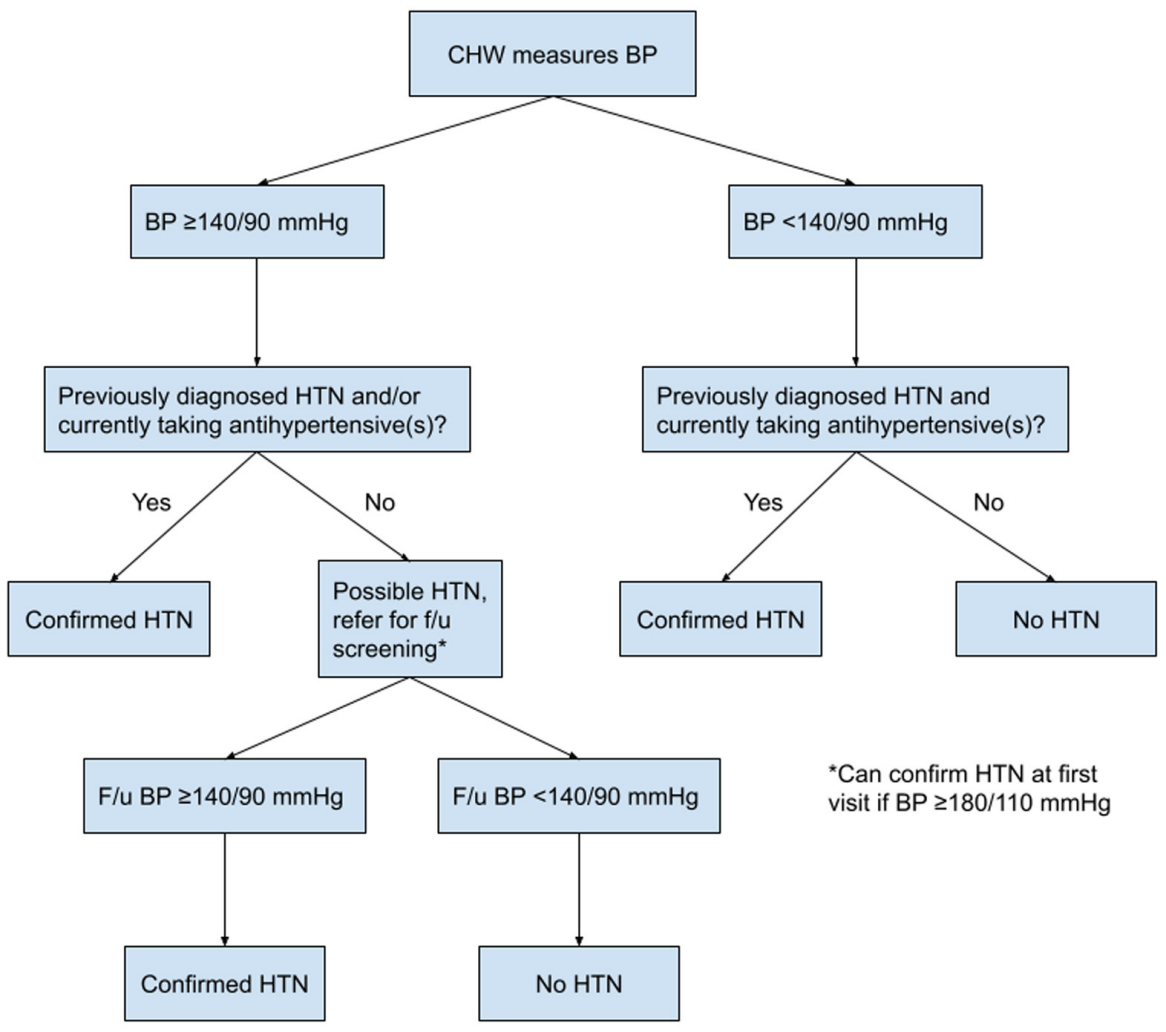
Hypertension diagnostic algorithm.

The diagnosis of hypertension by evaluators in this study was informed by guidelines from WHO (19) and the International Hypertension Society (IHS) (16). We developed a diagnostic algorithm for hypertension (Figure 1) based on these guidelines, which was incorporated in the mHealth application. The CHWs and physicians who participated in the study were trained on this algorithm.

We defined elevated systolic blood pressure (SBP) as ≥140 mmHg and elevated diastolic blood pressure (DBP) as ≥90 mmHg, consistent with international guidelines (16,19).

#### Measurement of blood pressure

BP was measured by CHWs and physicians using Omron M6 Comfort automatic upper arm BP monitors, a monitor that has been clinically validated by the STRIDE BP Initiative (22). Both evaluators used the same individual monitor to measure BP for each subject. BP was measured according to the protocols recommended by ISH and WHO guidelines (Table 1) (16,19). BP was measured one to three times for each subject: if the initial BP was ≥130/85 mmHg, two more measurements were taken at 1-minute intervals and the average of these two measurements was used.

**Table 1:** Standardized protocol for blood pressure measurement.


**1.** Prior to measurement, patient should have an empty bladder and not have smoked, drunk coffee, or exercised for 30 minutes

**2.** Patient sitting and relaxed for at least 5 minutes prior to the measurement

**3.** Cuff is placed on bare skin on the middle of the arm and maintained at the level of the heart during the measurement

**4.** During and between measurements: neither patient nor evaluator talks; back is supported; feet are maintained on the ground; legs are not crossed


#### Overview of hypertension screening protocol

Potential participants were first screened by a CHW for study eligibility. If a patient was eligible to participate and gave informed oral consent, the same CHW then collected basic demographic information, including name, date of birth (or self-reported age if the subject did not know exact date of birth), language, and community. Another CHW then conducted a hypertension screening with the assistance of the mHealth application, as described in the previous sections. At the conclusion of this assessment, the application provided a recommendation regarding the hypertension status of the patient based on information entered by the CHW: (1) no hypertension, (2) possible hypertension, or (3) confirmed hypertension. The category of possible hypertension accounted for patients who had an elevated blood pressure (≥ 140/90 mmHg), but no prior history of elevated blood pressure/hypertension, and so a confirmatory evaluation on a different date was required, as per international guidelines. The CHW then either affirmed this assessment or entered their own assessment if they disagreed with the application recommendation. The CHW did not share BP measurements or their assessment about hypertension status with the patient, nor with the physician who evaluated the patient next.

The evaluation process was then immediately repeated by the physician, facilitated by the mHealth application and using the same BP monitor that was used by the CHW to measure BP for each subject. At the conclusion of their evaluation, the physician informed each subject of their hypertension classification and provided direction regarding any follow-up care needed for blood pressure management. Those with possible hypertension were invited to return for a secondary blood pressure assessment occurring at least one week after this initial evaluation.

The secondary evaluation was carried out in a similar fashion to the initial evaluation, with paired, blinded assessments by a CHW and a physician facilitated by the mHealth application. Other than asking subjects about interim diagnosis of hypertension or prescription of antihypertensives by another medical provider, medical and social history were not repeated. At the conclusion of this secondary evaluation, CHWs and physicians were asked to assign each subject to one of two classifications: no current hypertension or confirmed hypertension.

### Statistical analysis

All statistical analyses were completed using R (23).

#### Analysis of diagnostic agreement (primary outcome)

The primary outcome for this study was agreement between the CHW and the physician on hypertension status. As there were three possible designations at the initial evaluation, we used a weighted Kappa approach to assign variable importance to how similarly CHWs assessed patients compared to the physician designation (24). The specific weighting scheme that we used was suggested by Cicchetti (25). In this scheme, there is greater disagreement weighting for more clinically concerning disagreement. We defined more clinically concerning disagreement as a misidentification of a patient as not having hypertension or possible hypertension when in fact they fell into one of these two categories, given the potential consequences of undiagnosed and untreated hypertension, including myocardial infarction, stroke, kidney disease, and heart failure (26). This decision resulted in the weighting scheme outlined in Table 2, which assigns lower weights to cases in which the CHW did not identify a patient with possible or confirmed hypertension.

**Table 2:** Weighting scheme for kappa analysis of CHW-physician agreement.


CHW ASSESSMENT	PHYSICIAN ASSESSMENT		

	NO	POSSIBLE	YES

No	1	1/3	0

Possible	1/3	1	2/3

Yes	0	2/3	1


Interpretation of the Kappa statistic varies, but the most widely accepted criteria are those postulated by Landis and Koch (24,27). We used a minimally acceptable criteria (MAC) (28) of 0.61, which corresponds to “substantial agreement” in this schema (27) and is advocated by McHugh as a minimum standard for clinical applications of the Kappa statistic (29). We calculated a one-sided 95% confidence interval for the weighted Kappa, testing for non-inferiority versus a null weighted kappa of 0.61, with the alternative hypothesis that the true weighted Kappa is <0.61. We established a threshold for statistical significance of ɑ = 5%.

Additionally, we calculated the overall accuracy of the CHWs’ assessment compared to physician assessment using percentage agreement with physician diagnosis (gold standard). In cases with completed follow-up screening, accuracy was assessed at each stage individually. While 80% is a widely-recommended MAC for percentage agreement (29), we used a higher standard of 90% based on previous studies of LHW hypertension and CVD risk screening (13,14). Similar to the analysis for Kappa, we generated a one-sided confidence interval to assess accuracy.

For cases in which CHW and physician assessment were discordant, we evaluated, based on the diagnostic algorithm, whether these differences could be attributed to differences in BP measurement alone, a combination of differences in recorded BP measurement and patient history (prior diagnosis of hypertension and/or current use of antihypertensives), or differences in patient history alone. For each of these cases, we also reported the direction of classification error for the CHWs (under or overdiagnosis). Finally, we calculated the percentage agreement of the CHWs and physicians with the patient classification provided by the application.

#### Secondary analyses

##### Bland-Altman analysis

We evaluated the agreement between blood pressure, weight and height measurements taken by the two evaluators using Bland-Altman analysis (24). We used the physicians’ measurements as a gold standard and compared it with the difference between physician and CHW measurements of each patient. The plots were created via the ‘blandr’ package (30). Bias was determined by whether the mean difference confidence interval contained the null value 0; if it contained 0, there was no significant bias shown.

##### CHW-Physician agreement on medical and social history and medication use

To evaluate the agreement between the physician and the CHW for intake of patient characteristics, we calculated Cohen’s Kappa for each variable. Due to the data skewing toward negative answers, we calculated and reported Michael’s Correlation Coefficient (MCC), Sensitivity, Specificity, Positive Predictive Value (PPV), and Negative Predictive Value (NPV), respectively, for each of the binary variables. Weighted Kappa was reported for continuous variables. Kappa was calculated via package ‘vcd’ (31), MCC via package ‘mltools’ (32), and sensitivity, specificity, PPV, and NPV were calculated via package ‘epiR’ (33).

##### Regression analysis

We implemented a logistic regression to evaluate how physician and CHW agreement on hypertension diagnosis was impacted by patient age, sex, and BMI (kg/m^2^), respectively. Continuous variables were centered and scaled. Odds Ratios and their 95% CI were reported.

#### Power analysis and sample size determination

Power was assessed to detect a weighted Kappa <0.61. We first used preliminary data from clinics conducted in the rural communities of SLT to estimate the anticipated percentages of patients falling into the no hypertension, possible hypertension, and hypertension categories upon initial physician assessment, which were 86%, 9%, and 5%, respectively. Various CHW agreement amounts were then assumed, with the remaining disagreement split between the other categories, with more assigned to one category difference of disagreement (e.g., CHWs have 80% agreement with no hypertension, but they indicate possible hypertension for 13.3%, and they indicate hypertension for 6.7%). The pairing of the estimated splits on initial physician designation, agreement/disagreement assignment, and the weighting matrix result in the alternative true weighted Kappa from which power was assessed.

Various agreement amounts and sample size combinations were examined. In each combination, 10,000 simulated data sets were assessed, with power estimated to be the proportion of times the inferior weighted Kappa was detected. One-sided confidence intervals described above tested the null hypothesis that Kappa = 0.61. With a sample size of at least 350 patients initially screened, we calculated >80% power to detect an inferior weighted Kappa difference of 0.13. This corresponded to a CHW accuracy of 80%, with >90% power to detect this inferior accuracy versus a null accuracy of 90%.

## Results

### Participants

**Figure 2:**
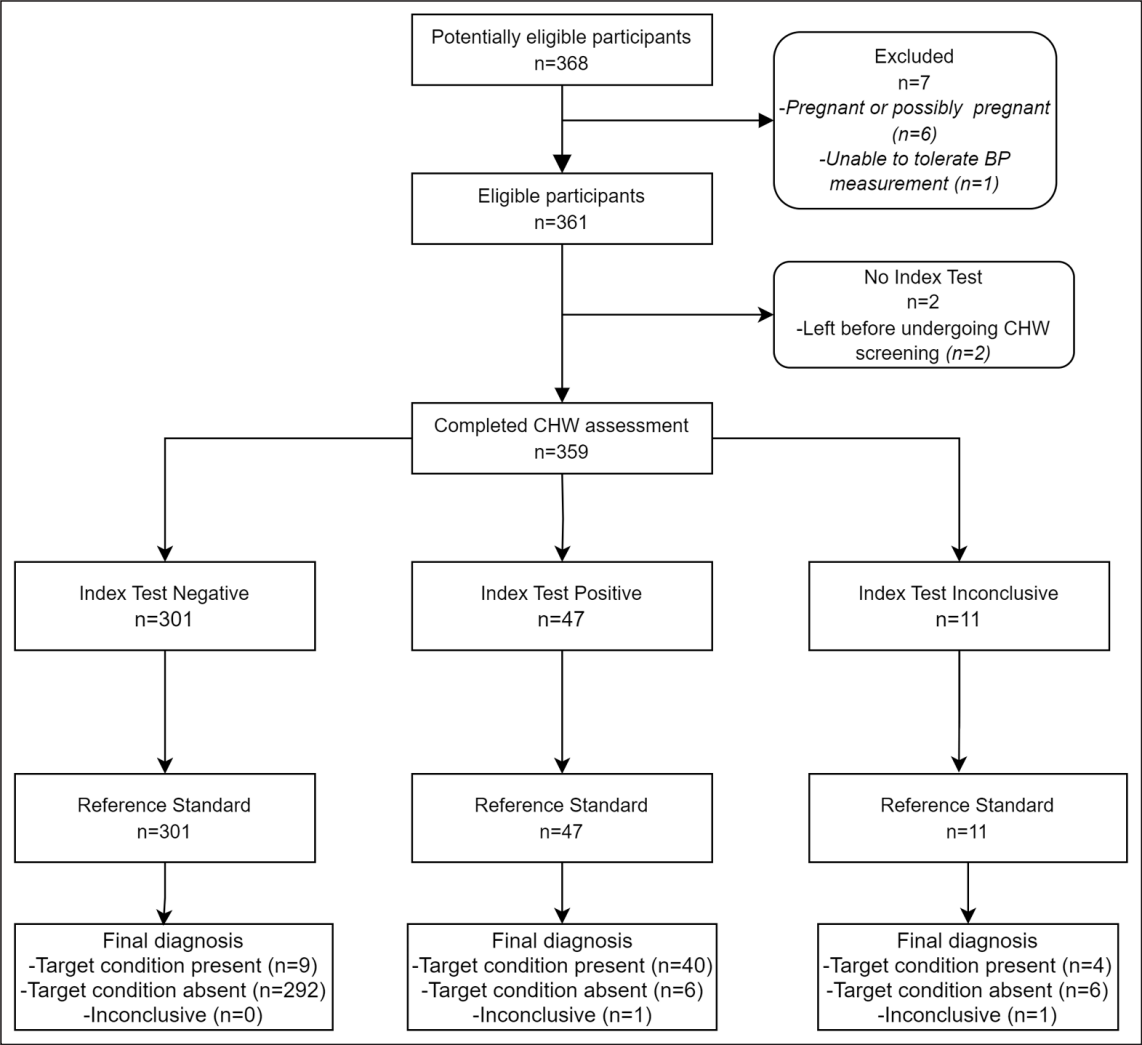
Participant flow diagram and final diagnoses.

Figure 2 outlines participant flow throughout the study. 359 subjects underwent primary screening by a CHW and a physician. Table 3 summarizes demographic characteristics for the sample as a whole as well as for subject subgroups by initial hypertension categorization (per physician assessment). Of note, subjects identified as having hypertension or possibly hypertensive were significantly older and more likely to have diabetes than normotensive subjects.

**Table 3:** Demographic characteristics of study subjects by hypertension classification at primary screening.


	ALL SUBJECTS	NO HTN*	POSSIBLE HTN	CONFIRMED HTN	*P* OVERALL

	*N* = 359	*N* = 302	*N* = 13	*N* = 44	

Age, years (SD)	45.9 (16.1)	43.9 (15.8)	58.8 (14.3)	55.3 (13.1)	<0.001

Sex, n (%):					0.071

Female	298 (83.0%)	255 (84.4%)	8 (61.5%)	35 (79.5%)	

Male	61 (17.0%)	47 (15.6%)	5 (38.5%)	9 (20.5%)	

Physician measured SBP, mmHg (SD)	116 (18.5)	111 (11.9)	149 (11.6)	144 (22.7)	<0.001

CHW measured SBP, mmHg (SD)	114 (19.1)	109 (13.1)	144 (11.3)	140 (25.6)	<0.001

Physician measured DBP, mmHg (SD)	73.8 (9.74)	71.4 (7.52)	84.0 (8.65)	86.8 (11.4)	<0.001

CHW measured DBP, mmHg (SD)	73.8 (10.1)	71.9 (8.44)	82.2 (9.01)	84.0 (13.2)	<0.001

Body mass index, kg/m^2^ (SD)†	29.2 (5.55)	29.1 (5.52)	31.0 (9.08)	29.1 (4.37)	0.489

History of diabetes, n (%):	43 (12.0%)	25 (8.28%)	5 (38.5%)	13 (29.5%)	<0.001

Number of days per week with physical activity (SD)	4.42 (3.07)	4.46 (3.05)	3.38 (3.28)	4.41 (3.13)	0.464


*Patient categorization (no HTN, possible HTN, confirmed HTN) reported based physician categorization. †BMI, history of diabetes, and physical activity days reported for physician assessment.

Of the 13 subjects identified as possibly having hypertension at primary screening, 11 underwent secondary screening and two were lost to follow-up.

### Primary outcome – Hypertension diagnostic accuracy of CHWs compared to physician

#### CHW diagnostic accuracy at the initial (primary screening) visit

Table 4 provides a cross tabulation of patient hypertension classification by CHWs and physicians for the first diagnostic screening visit.

**Table 4:** Cross tabulation of CHW and physician hypertension assessment at primary screening visit.


CHW ASSESSMENT	PHYSICIAN ASSESSMENT			

	NO	POSSIBLE	YES	TOTAL

No	**292**	1	7	300

Possible	6	**9**	4	19

Yes	4	3	**33**	40

Total	302	13	44	359


*Note:* Bolded numbers in represent cases of complete agreement between physician and CHW.

We found substantial agreement with patient hypertension assessment during the initial visit among physicians and CHWs (weighted Kappa = 0.8, 95% CI = 0.72, 0.88) and cleared the MAC of Kappa = 0.61, meaning that the CHWs are not inferior to physician assessment. As a sensitivity analysis, we also calculated unweighted Kappa, which also cleared this MAC (Kappa = 0.75, 95% CI = 0.67, 0.84). Calculated overall diagnostic accuracy for CHWs was 93% (95% CI = 90.4%, 95.7%), which was also higher than the pre-specified MAC of 90%.

Of the 25 discordant cases, differences in categorization between CHWs and physicians could be attributed to differences in BP measurements for 13 cases (52.0%), differences in BP measurements and patient history (prior diagnosis of hypertension and/or current use of antihypertensives) in 5 cases (20.0%), and differences in history alone in 7 cases (28.0%). Under- and over-diagnosis by CHWs compared to physicians for these cases were evenly divided, with 13 cases (52.0%) of underdiagnosis and 12 cases (48.0%) of overdiagnosis. Likewise, discrepancies attributable to differences in BP measurement alone were evenly divided, with 6 cases (46.2%) of CHW underdiagnosis and 7 cases (53.8%) of CHW overdiagnosis.

#### CHW diagnostic accuracy at the follow-up (secondary screening) visit

Table 5 outlines physician and CHW agreement for the 11 patients who completed the secondary screening visit. Hypertension diagnostic agreement between CHWs and physicians was 63.6% at this visit. For all 4 discordant cases, differences in BP measurements between CHWs and physicians accounted for the discrepancy in categorization. Half of these cases represented CHW underdiagnosis and half overdiagnosis. Due to the low number of patients undergoing secondary screening, no further statistical analysis was conducted.

**Table 5:** Cross tabulation of CHW and physician hypertension assessment at secondary screening visit.


CHW ASSESSMENT	PHYSICIAN ASSESSMENT		

	NO	YES	TOTAL

No	**0**	2	2

Yes	2	**7**	9

Total	2	9	11


*Note:* Bolded numbers in represent cases with agreement between physician and CHW.

#### Overall CHW diagnostic accuracy and agreement with application classification

Table 6 is a cross tabulation of overall hypertension classification by physicians and CHWs, accounting for final diagnosis for patients who underwent secondary screening. Overall CHW diagnostic accuracy was 92.8% (95% CI = 90.1%, 95.4%), higher than the pre-specified MAC of 90%. Of the 370 patient encounters, the CHWs agreed with the hypertension classification suggested by the application in all cases, while physicians agreed with this classification in all but 2 cases (99.5%).

**Table 6:** Cross tabulation of overall CHW and physician hypertension assessment.


CHW ASSESSMENT	PHYSICIAN ASSESSMENT			

	NO	POSSIBLE	YES	TOTAL

No	**292**	0	9	301

Possible	6	**1**	4	11

Yes	6	1	**40**	47

Total	304	2	53	359


*Note:* Bolded numbers in represent cases of complete agreement between physician and CHW.

#### Regression analysis

At the 0.05 level, there was no statistically significant impact of patient age, sex, or BMI on agreement between the CHWs and physicians for hypertension diagnosis, as assessed by univariable and multivariable logistic regression. There was a trend towards statistical significance for the effect of age on CHW agreement: for a 5-year difference in patient age, the odds of CHW agreement with the physician on hypertension diagnosis is 11% lower (OR = 0.89, 95% CI = (0.78, 1.02), p = 0.09).

### Secondary outcomes

#### Bland-Altman analysis of measurement agreement between CHWs and physicians

For Bland-Altman analysis, we determined bias in measurements by whether the mean difference confidence interval contained the null value 0; if it contained 0, there was no significant bias shown. Three measures were shown to have statistically significant bias: SBP, height, and BMI.

**Figure 3:**
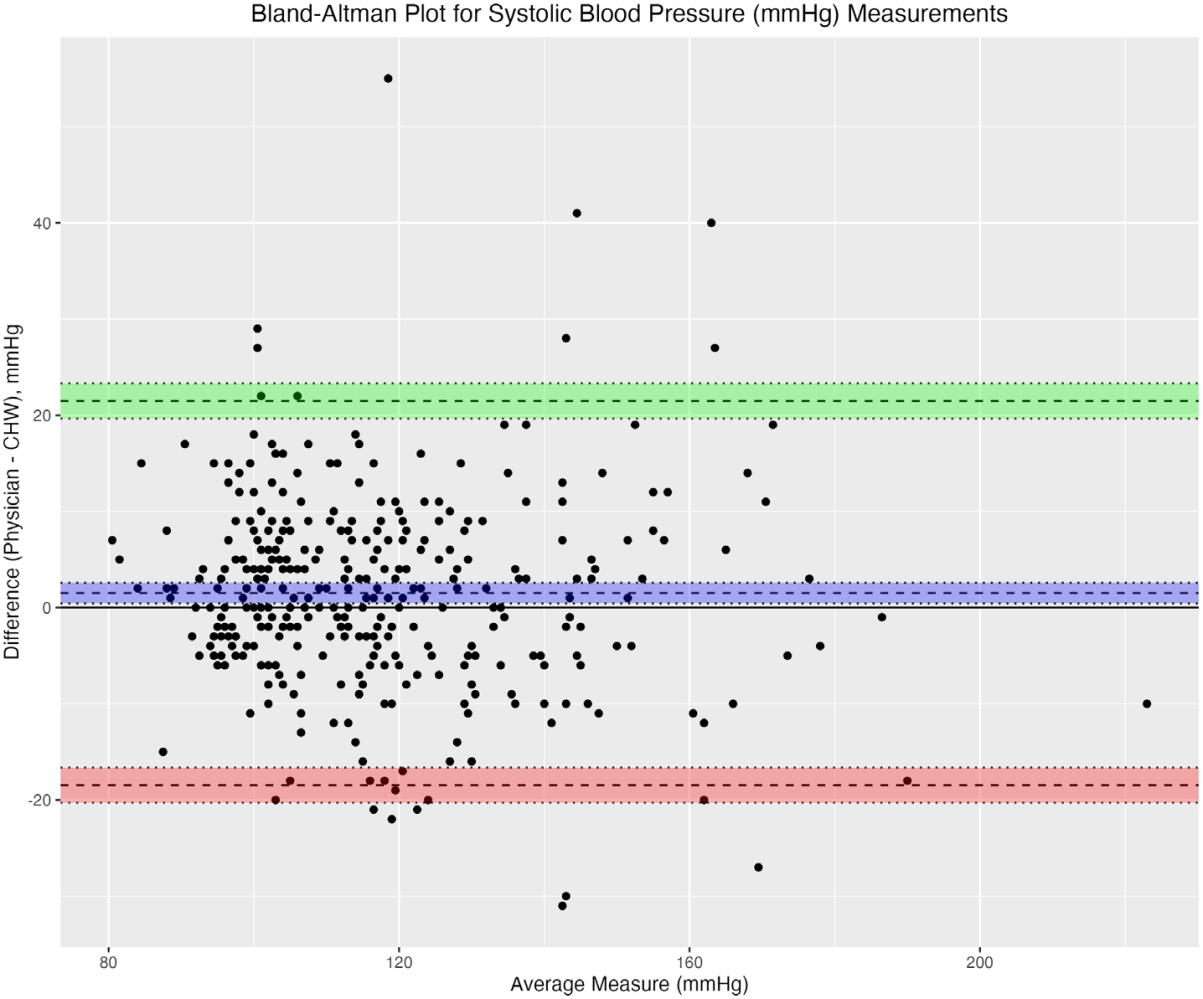
Bland-Altman plot for systolic blood pressure (mmHg) measurements.

As shown in Figure 3, a Bland-Altman plot of SBP measurements, CHWs tended to record lower SBP measurements as compared to physicians, though the mean difference (dotted line with purple confidence interval) was close to 0 (1.51 mmHg, 95% CI 0.44 to 2.58). This trend was most pronounced when the average of CHW and physician SBP was greater than 120 mmHg.

**Figure 4:**
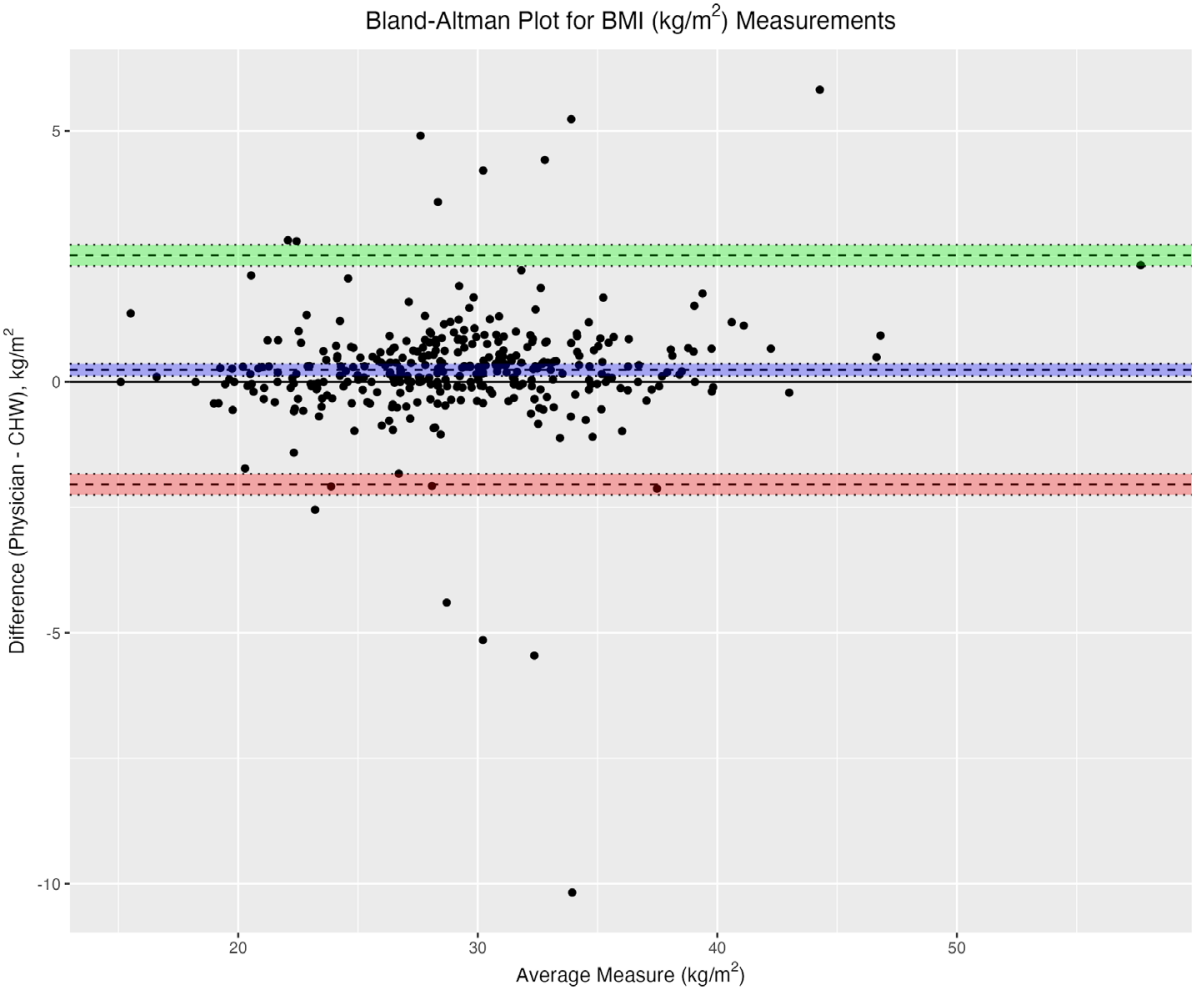
Bland-Altman plot for BMI (kg/m^2^) measurements.

BMI calculations (Figure 4) based on height and weight measurements taken by CHWs tended to be lower than those calculated from physician measurements (0.24 kg/m^2^, 95% CI 0.12 to 0.36). This was attributable to significant bias in CHW height measurements to greater heights, as BMI and height are inversely related, and weight measurements were not found to be biased between groups.

#### CHW and physician agreement on past medical history, medication use, and social history elements

Most patient characteristics showed moderate (Kappa: 0.41–0.6), substantial (Kappa: 0.61–0.8), or almost perfect (Kappa: 0.81–1) agreement between physician and CHW (Tables 7, 8, 9). Due to low prevalence of most patient characteristics, CHW specificity and NPV were nearly perfect for all metrics. CHWs had substantial sensitivity and PPV for several characteristics, including history of diabetes, current use of antihypertensive medication, and whether the patient had smoked or drank alcohol beverages recently. Patient history elements with lower agreement between CHWs and physicians included CAD, heart failure, CKD, and physical activity levels.

**Table 7:** CHW and physician agreement on patient characteristics (binary variables).


	PHYSICIAN IDENTIFIED POSITIVE	OVERALL AGREEMENT	KAPPA	MCC	SENSITIVITY	SPECIFICITY	PPV	NPV

**Coronary Artery Disease (CAD)**	1 (0.28%)	96.94%	–0.01	–0.01	0.00	1.00	0.00	0.97

**Heart Failure**	5 (1.39%)	98.05%	–0.01	–0.01	0.00	0.99	0.00	0.99

**Diabetes**	43 (11.98%)	98.61%	0.93	0.93	0.95	0.99	0.93	0.99

**Stroke**	8 (2.23%)	96.94%	0.51	0.53	0.40	0.99	0.75	0.97

**Hyperlipidemia**	30 (8.36%)	94.15%	0.57	0.58	0.70	0.96	0.53	0.98

**Chronic Kidney Disease (CKD)**	9 (2.51%)	94.99%	0.33	0.36	0.26	0.99	0.56	0.96

**Hypertension**	93 (25.91%)	89.14%	0.71	0.71	0.81	0.92	0.75	0.94

**Taking Antihypertensive Medication**	26 (7.24%)	98.33%	0.87	0.87	0.92	0.99	0.85	0.99

**Smokes**	12 (3.34%)	99.72%	0.96	0.96	1.00	1.00	0.92	1.00

**Drinks Alcohol (30 Days)**	27 (7.52%)	98.05%	0.86	0.86	0.88	0.99	0.85	0.99


**Table 8:** CHW and physician agreement on patient characteristics (continuous and categorical variables).


	KAPPA

**Stove Type**	0.58

**Cooking Location**	0.48

Low Activity (7 days)*	0.24

High Activity (7 Days)*	0.25


*Note:* *Weighted Kappa reported for continuous variables.

**Table 9:** CHW and physician agreement on patient characteristics (continuous and categorical variables).


	TYPICAL # CIGARETTES/DAY	# ALCOHOLIC DRINKS LAST 30 DAYS

**Subset**	0.4 | 0.63	0.21 | –0.04

**All participants (N = 359)**	0.7 | 0.86	0.66 | 0.73


*Note:* Kappa reported as: “Unweighted Kappa | Weighted Kappa”. Subset of smokers N = 12Subset of alcohol drinkers N = 30.

In terms of antihypertensive medication use, CHWs showed high agreement with physicians for names (Kappa = 0.82) and daily dosages (Kappa = 0.72) of medications reported by patients (Table 10). Kappa did decrease when limiting analysis to only patients who reported taking at least one antihypertensive medication (Kappa = 0.57 and 0.44 for medication name and daily dosage, respectively).

**Table 10:** CHW and physician agreement on antihypertensive medication use.


	MEDICATION NAMES	DAILY DOSAGE (MG)

**All Subjects (N = 1077)**	0.82	0.72

**Subjects with at least one medication listed; 3 medications (N = 84)**	0.76	0.64

**Subjects with at least one medication listed (N = 36)**	0.57	0.44


*Note:* Subjects with at least one medication listed (N = 84) includes 3 observations per patient. Subjects with at least one medication listed (N = 36) includes only observations with a medication listed.

## Discussion

In this blinded diagnostic study of 359 patients in rural Guatemala, we found that CHWs using an mHealth tool can screen patients for hypertension with similar accuracy to a physician. Diagnostic agreement between CHWs and physicians was higher than predefined minimally acceptable criteria (MAC) using both weighted Kappa (0.8 vs MAC of 0.61) and overall proportional agreement (93% vs MAC of 90%). Subject age, sex, and BMI did not have a statistically significant impact on CHW and physician agreement on hypertension diagnosis, though there was a trend towards significance for the effect of age (lower odds of agreement in older patients). Agreement was also high between CHWs and physicians for most elements of the patient history, including prior history of hypertension or diabetes, current use of antihypertensive medication, and recent smoking and alcohol use. Agreement on other history components, such as prior history of CAD or heart failure and physical activity, was less optimal. Finally, there were small but statistically significant differences in SBP and BMI measurements, with CHWs recording lower SBP measurements and lower BMI measurements than physicians.

CHWs in this study had similar diagnostic accuracy for hypertension to that reported for LHWs in previously published studies. John et al. (12) trained LHWs working in rural India to manually measure blood pressure. These LHWs then screened 920 adults between 50 and 80 years old for elevated blood pressure, of whom 20% were also screened by a blinded investigator. Weighted Kappa for categorical agreement in blood pressure readings (normal, prehypertensive, or hypertensive range) between LHW and investigator was high, ranging from 0.62 to 0.89. Similarly, Teshome et al. trained health extension workers to manually measure blood pressure in rural Ethiopia (34). These workers then measured BP for 1,177 people, and these assessments were paired with BP measured by a health professional. Inter-rater agreement for high blood pressure detection was high (Kappa = 0.912). Of note, assessment of agreement in these studies was based on categorization of blood pressure measurements alone, not diagnosis of hypertension (as in our current study), which also requires incorporation of medical history and medication use and is thus inherently more complex. Additional strengths of our study compared to previous studies were the use of physicians as a gold standard comparator and *a priori* definition of acceptable Kappa value for inter-rater agreement.

As with blood pressure characterization, our results compare favorably to the existing literature on the reliability of LHWs to discern cardiovascular risk. Abegunde et al. (13) compared CVD risk assessment performed by LHWs and physicians in India and Pakistan using algorithms from the WHO Cardiovascular Risk Management Package. They analyzed concordance between 649 paired evaluations by an LHW and a physician, who were appropriately blinded to each other’s assessment, finding agreement greater than the *a priori* level of 80% for blood pressure and demographic factors contributing to CVD risk. Kappa statistics for LHW/physician agreement on CVD risk factors were similar or improved in our study compared to Abegunde et al. (13) for diabetes (0.93 vs 0.83), stroke (0.51 vs 0.33), tobacco use (0.96 vs 0.74), and alcohol use (0.86 vs 0.77). While Abegunde et al. (13) reported superior Kappa statistics for history elements suggestive of CAD (heart attack with Kappa = 0.80 and angina with Kappa = 0.62) than our study (Kappa = –0.01 for history of CAD), this can largely be seen as an artifact of very low reported prevalence in our patient population. Though overall proportional agreement between CHWs and physicians for CAD was very high (96.9% compared to 99.5% for heart attack and 90.2% for angina in Abegunde et al. (13)), only one patient out of 359 in our cohort was identified by the physicians as having a history of CAD.

Abegunde et al. (13) demonstrated that LHWs could risk stratify patients with comparable accuracy to a physician with only three additional days of training, which strengthened the generalizability of their approach in low resource settings. In this study, CHWs received only 12 hours of additional training and yet achieved similar accuracy. We hypothesize that the use of an mHealth tool providing clinical decision support played a key role in achieving this level of performance with less training time. This is consistent with our experience implementing other mHealth programs with CHWs in rural Guatemala and with the growing evidence base for mHealth tools designed for LHWs in LMIC (9,11).

An additional strength of this study was the use of validated automated oscillometric BP measuring devices, the current recommended global standard of care for hypertension screening and management (16,35), and comparison of measurements by CHWs and physicians using the same machine for each subject. Abegunde et al. (13), John et al. (12) and Teshome et al. (34) all found that LHWs could manually measure BP with comparable accuracy to physicians and other health professionals, such as nurses. Supportive of current guidelines advocating the use of automated BP measurement, Reidpath et al. (15) found that lay people using automatic devices were more accurate in BP measurement than qualified health workers using manual devices. However, we could find no other studies in the published literature comparing automatic BP measurement by LHWs and physicians.

Bland-Altman analysis showed a slight bias in SBP measurements between CHWs and physicians in this study, with mean SBP 1.51 mmHg lower for CHWs, and no significant difference in DBP. Higher SBP measurements for physicians compared to CHWs, even when using the same automatic BP measuring device and technique, could reflect the “white coat effect.” This effect has previously been implicated as an explanation for the difference between home BP measurements or unattended (e.g., fully automated) BP measurements in a clinical setting and measurements taken by medical personnel, which tend to be higher than the former (36). Our finding of lower SBP measurements for CHWs compared to physicians is consistent with previous studies reporting lower BP measurements for nurses compared to physicians (37). In this way, our study adds to the literature suggesting that use of non-physician health workers in BP measurement can mitigate the white coat effect and potentially prevent overdiagnosis of hypertension, with the caveat that differences in BP measurements between CHWs and physicians were equally likely to lead to overdiagnosis or underdiagnosis.

The difference in height measurements between CHWs and physicians was statistically significant, but not likely clinically significant (mean difference 0.62 cm higher for CHWs than physicians). As there were no statistically significant differences in weight measurements, the small difference in BMI (mean difference 0.24 kg/m^2^ lower for CHWs), was attributable to higher mean height measurements in CHWs. Differences in height measurements may be due to differences in technique, as the CHWs generally had more experience with measuring patient heights due to their extensive work in a childhood nutrition program in these communities.

One potential limitation of our approach was that the mHealth applications used by CHWs and physicians in this study provided essentially the same clinical decision support. Therefore, it is possible that this guidance biased both evaluators toward the same diagnostic classification, particularly as there was high agreement with application classification on the part of both CHWs (100%) and physicians (99.5%). We mitigated this limitation by developing the diagnostic algorithms in concert with the evaluating physicians so that they reflected both standardized guidance as well local practice and the clinical reasoning of the evaluators. In addition, both CHWs and physicians were encouraged to use their own judgment with regard to hypertension diagnostic classification and told that the clinical decision support was only advisory. Finally, the lack of variation in the order of diagnostic assessments (CHW assessment always first, followed by physician assessment) could have introduced a systematic bias into the results. We felt that varying the order of assessments was not logistically feasible and took measures to limit the risk of bias by blinding CHWs and physicians to each other’s assessments and rigorously standardizing the measurement of blood pressure by each assessor, including ensuring that subjects were resting for an adequate amount of time before blood pressures were measured.

## Conclusion

This study demonstrates that CHWs supported by mHealth can identify patients with hypertension, as well as other cardiovascular risk factors, with similar accuracy to a physician. This approach can be adapted to other low-resource settings around the world to help decrease the burden of unidentified and untreated hypertension and could also be helpful even in higher resource settings to offload physicians and other advanced practitioners, allowing them to focus on more complex clinical tasks. We plan to freely share the application designed for this study CommCare Application Library to facilitate such adaptation. Future studies should evaluate implementation of LHW hypertension and cardiovascular risk screening at scale, assessing population impact on cardiovascular morbidity and mortality, as well as test approaches involving LHWs in hypertension management as well as screening and diagnosis.

## Data Accessibility Statement

The datasets generated and/or analyzed during the current study are not publicly available as such sharing outside of the research team was not part of the research protocols approved by ethical oversight committees for this project, but may be made available upon reasonable request from the corresponding author with IRB permission.

## Additional File

The additional file for this article can be found as follows:

10.5334/gh.1423.s1 Appendix 1. STARD Checklist.
